# The Impact of Surgical Boot Camp and Subsequent Repetitive Practice on the Surgical Skills and Confidence of Residents

**DOI:** 10.1007/s00268-020-05669-x

**Published:** 2020-07-06

**Authors:** Wei Wang, Hucheng Ma, Haozhen Ren, Zhongxia Wang, Liang Mao, Ningning He

**Affiliations:** Department of General Surgery, Nanjing Drum Tower Hospital, The Affiliated Hospital of Nanjing University Medical School, No. 321 Zhongshan Road, Nanjing, 210008 Jiangsu People’s Republic of China

## Abstract

**Background:**

Boot camp can enable residents to acquire surgical skills and confidence, but they can lose these skills over time if they do not use them. The purpose of this study was to explore whether boot camp and subsequent repetitive practice could strengthen residents’ clinical skills and self-confidence.

**Methods:**

This is a comparative study of surgical residents who were enrolled in our institution from 2016 to 2017. The residents in the experimental group (enrolled in 2017) received boot camp training and a year of repetitive practice. The control group (enrolled in 2016) only received routine residency training. The rotation assessment pass rates of the two groups during the first year of the residency training were compared. A survey was conducted at different points in time to investigate the influence of boot camp and repetitive practice on the confidence of the residents.

**Results:**

The assessment pass rate of the experimental group was significantly higher than that of the control group (*p* < 0.05). The residents’ confidence in themselves improved significantly after the boot camp, and it was comparable to that of the residents in the control group after their first year of residency. The level of self-confidence of the experimental group was further improved after repetitive practice. Finally, residents in the experimental group received better evaluations by their colleagues than the control group received.

**Conclusions:**

This study showed that boot camp can improve the surgical skills and confidence of residents and that repetitive practice can further strengthen them. Residents in the experimental group developed their self-confidence in boot camp, and it increased after repetitive practice.

## Introduction

The National Health Commission of the People’s Republic of China issued guidelines in 2014 on the establishment of a standardized training system for residents, marking the beginning of residency training in China [1]. Although medical graduates must pass standardized examinations before becoming residents, there is some variability in their knowledge and skills. Not everyone who starts a residency training program has the appropriate technical or non-technical skills to take advantage immediately of learning opportunities at work or has the confidence in their skills to be assertive within the team. As a result, the cumulative effect of these factors may adversely affect the self-confidence of these recruits and even their patients’ safety [2].

An alternative strategy to solve this problem is to deliver an intensive curriculum in a short time just prior to beginning residency. Residency boot camp, similar to military boot camp, may serve as an effective approach for medical graduates to adapt to the transition from student to resident as soon as possible [3]. The American Surgical Association’s Blue Ribbon Committee recommended, as early as 2004, that “surgery departments should strive to make surgical clerkship and resident preparedness courses of the highest quality” [4]. In 2015, the American Board of Surgery, American College of Surgeons, Association of Program Directors in Surgery, and the Association for Surgical Education publicly released the jointly developed Resident Prep Curriculum [5].

Residency boot camps have not been widely implemented in China. Although most residents receive pre-career education before entering their training programs, this kind of education often emphasizes information about hospital rules. They fail to recognize the assessment of professional competence, and the homogeneous training medical students receive may lead to trainees to be at different starting points on the first day of residency. This is not conductive to the implementation of standardized resident training and the objective evaluation of training effectiveness. Our institution has held a two-week surgical boot camp since 2017 to solve these problems. Residents also were required to participate in the multiple training days during the first year after the boot camp. The purpose of this study was to investigate the effects of this training program on novice residents’ confidence in their ability to perform, and their actual performance on surgical tasks.

## Material and methods

### Participants

The study was a retrospective comparison of the results of sequential years of training conducted using two different methods. The research sample was surgical residents enrolled in our institution from 2016 to 2017. The residents enrolled in 2017 were assigned to the experimental group. The program for the experimental group was a boot camp that consisted of a formal program to teach essential technical and non-technical skills, which were then reinforced by continued formal practice sessions repetitive over the year. The residents in the control group (enrolled in 2016) did not participate in the boot camp training or repetitive practice, but they received a similar residency training program throughout the year and completed a year of workplace experience in our hospital. Data on characteristics of the participants, including age, gender, academic degree, and specialty, were collected. This study was approved by the ethical and scientific committee of our institution, and oral informed consent was obtained from all the participants.

### Intervention

#### Surgical boot camp

The boot camp was two weeks long, and the training courses were provided from Monday to Friday. The training content, which was core competency-oriented, included basic medical theory, basic surgical skills, management of surgical complications, and communication skills. The boot camp curriculum was developed according to the guidelines for residency training released by the National Health and Family Planning Commission (NHFPC) in 2014 [6] and is shown in "Appendix" Table 1. The training methods included didactic lecture, simulation, case-based learning, an animal laboratory, and teaching ward rounds.

**Table 1:** The characteristics of the residents

	Experimental group	Control group	*p*
Age (years)	27(25–33)	27(25–32)	0.68
Gender			0.49
Male	27	21	
Female	1	3	
Educational background			0.25
BS	3	6	
MM	13	12	
MD	12	6	
Specialty			0.67
General surgery	11	6	
Orthopedics	3	4	
Urology	6	3	
Neurosurgery	4	4	
Cardiothoracic surgery	3	5	
Plastic surgery	1	2	

BS: Bachelor's degree; MM: Master of Medicine; MD: Doctor of Medicine

#### Repetitive practice

Residents in experimental group participated in repetitive practice for one year after the boot camp. The content of the repetitive practice was mainly focused on what the residents had learned during boot camp. They were required to have at least 5 h of skills training per week, and the total training time was not less than 30 h per month. Training was usually done after the residents had completed their daily work in the hospital or during their leisure time.

### Measures and comparisons

#### Department rotation assessment

Department rotation assessments were done in both the experimental group and the control group. Residents had to pass the rotation assessment, which included a written examination and a simulated operation, when they completed the rotation of a surgical department. The content included medical theoretical knowledge and the basic skills for each professional department. Table 2 in the “Appendix” shows the scoring criteria of a simulated operation. The total time residents participated in assessments (people-time), and the total people-time of those who passed the assessments within one year were counted.

**Table 2:** Comparison of the rotation assessment results of the two groups

	Pass	Fail	Total
Experimental group	95	11	106
Control group	79	21	100
Total	174	32	206
*p*	0.04		

#### Self-confidence

Residents' confidence to cope with routine medical work was assessed through questionnaires. The content of the questionnaire included communication skills, management of surgical patients, basic surgical operations, etc. The format of questionnaire was based on a previous study [7]. All the residents were asked to rate their comfort level on a scale ranging from 1 (not comfortable) to 5 (very comfortable).

Evaluation by colleagues. The staff of a department who had working contact with a resident were surveyed for the same questions when the resident completed training in that department. Each resident was rated by 3 doctors and 2 nurses, using a scale from 1 (very bad) to 5 (very good).

### Statistical analysis

The data were analyzed using SPSS 20.0 (IBM Corporation, Armonk, NY). Paired *t* tests were used to compare the data from the residents in the experimental group at two points in time. Two-sample *t* tests were used to compare the experimental group with the control group. Chi-square tests were used to compare differences in the proportions of the two groups on different variables. A *p* value < 0.05 was considered statistically significant.

## Results

### Characteristics of the residents

A total of 52 residents were included in this study. Among them, 28 residents were in the experimental group and the remaining 24 residents were in the control group. The characteristics of all the residents are shown in Table 1. There were no significant differences between the general characteristics of the two groups.

### Department rotation assessment

Our results showed that residents in the experimental group received a total of 106 department rotation assessments in 2017, and their overall pass rate was 89.62% (95/106). The pass rate of the control group was 79.00% (79/100) in 2016. The performance of the experimental group was significantly better than the control group (*p* < 0.05) (Table 2).

### Self-confidence

The survey of residents’ confidence to deal with routine medical tasks was conducted before the surgical boot camp and one year after it. The same survey also was conducted with the control group at the end of 2016. Our results (Table 3) showed that the confidence of residents in the experimental group improved significantly after the boot camp, except for two skills: “handle daily medical record writing (question 3)” and “perform a surgical puncture (question 9).” After one year of repetitive practice, the scores on all the items were significantly higher compared to those after the boot camp.

**Table 3:** The survey results of residents’ confidence to deal with routine medical tasks

Questions	*A*	*B*	*C*	*D*	*B* vs *A*	*B* vs *D*	*C* vs *B*	*C* vs *D*
*Non-surgical skill*								
1	2.86(1.04)	3.61(0.63)	3.96(0.69)	3.63(0.82)	< 0.01	0.93	< 0.01	0.11
2	2.96(1.04)	3.50(0.51)	3.86(0.65)	3.83(0.70)	< 0.05	0.05	< 0.01	0.90
3	3.61(1.06)	3.71(0.81)	4.00(0.94)	4.17(0.70)	0.52	< 0.05	< 0.05	0.47
4	2.94(0.96)	3.75(0.65)	4.04(0.74)	3.96(0.75)	< 0.01	0.29	< 0.05	0.71
5	3.07(0.94)	3.71(0.60)	4.07(0.67)	3.71(0.75)	< 0.01	0.97	< 0.01	0.07
6	2.93(0.98)	3.50(0.58)	3.89(0.83)	3.38(0.65)	< 0.01	0.47	< 0.01	< 0.05
7	3.00(0.90)	3.79(0.69)	4.21(0.67)	3.75(0.74)	< 0.01	0.86	< 0.01	< 0.05
8	2.79(0.99)	3.57(0.74)	3.96(0.88)	3.96(0.75)	< 0.01	0.07	< 0.01	0.97
*Surgical skills*								
9	3.11(1.10)	3.36(0.56)	3.64(0.78)	3.38(0.77)	0.26	0.92	< 0.05	0.22
10	3.18(0.90)	3.61(0.69)	3.96(0.74)	3.96(0.81)	< 0.05	0.09	< 0.05	0.98
11	3.32(0.90)	3.68(0.67)	4.11(0.74)	4.13(0.68)	0.05	< 0.05	< 0.01	0.93
12	2.86(0.89)	3.61(0.63)	4.07(0.72)	3.54(0.72)	< 0.01	0.73	< 0.01	< 0.05
13	2.82(0.98)	3.79(0.63)	4.18(0.67)	4.38(0.49)	< 0.01	< 0.01	< 0.01	0.24
14	2.86(0.93)	3.86(0.65)	4.43(0.57)	4.00(0.72)	< 0.01	0.46	< 0.01	< 0.05
15	2.54(1.04)	4.04(0.74)	4.29(0.66)	4.00(0.66)	< 0.01	0.86	< 0.05	0.13
16	2.64(0.91)	3.89(0.69)	4.25(0.70)	3.88(0.79)	< 0.01	0.93	< 0.01	0.08
17	2.71(0.98)	3.68(0.61)	4.36(0.68)	3.96(0.81)	< 0.01	0.16	< 0.01	0.06
18	2.96(1.14)	3.96(1.14)	4.32(0.61)	3.88(0.74)	< 0.01	0.64	< 0.01	< 0.05
19	3.00(0.77)	3.54(0.74)	3.89(0.83)	3.54(0.78)	< 0.05	0.98	< 0.01	0.12
20	2.64(0.95)	3.43(0.57)	3.82(0.77)	3.96(0.69)	< 0.01	< 0.01	< 0.01	0.51
21	2.96(0.92)	3.71(0.53)	4.18(0.67)	3.42(0.78)	< 0.01	0.11	< 0.01	< 0.01

1. Smooth communication with patients; 2) work well with colleagues; 3) handle daily medical record writing; 4) prescribing preoperative prescriptions for different operations; 5) prescribing postoperative fluids; 6) differential diagnosis and basic management of an acute abdomen problem; 7) management of trauma patients; 8) management of patients with surgical complications; 9) perform a surgical puncture; 10) do a simple resection of superficial lesion; 11) place patient in correct surgical position; 12) deciding on the incision to be used in an abdominal surgery; 13) define the scope of disinfection for different operations; 14) determining the margins when excising a lesion; 15) naming surgical instruments correctly; 16) doing a one-handed reef knot; 17) doing an instrument tie; 18) doing a vessel ligation at depth; 19) doing a simple suture on my own; 20) deciding on the position of trocar during laparoscopic surgery; 21) navigating a camera during laparoscopy

*A*: Pre-boot camp, mean (standard deviation, SD); *B*: post-boot camp, mean (SD); *C*: annual survey of experimental group, mean (SD); *D*: control group’s annual survey, mean (SD)

The comparison between the experimental group and control group showed that the confidence of residents who completed boot camp training was comparable to that of the control group who had completed a year of residency training. Furthermore, after a year of repetitive practice, the residents in the experimental group had significantly higher confidence in their skills related to “differential diagnosis and basic management of an acute abdomen problem (question 6),” “management of trauma patients (question 7),” “deciding on the incision to be used in an abdominal surgery (question 12),” “determining the margins when excising a lesion (question 14),” “doing a vessel ligation at depth (question 18),” and “navigating a camera during laparoscopy (question 21)” (*p* < 0.05).

### Evaluation by colleagues

The survey of residents' colleagues showed that residents in the experimental group were rated higher on all the items than were those in the control group. The residents’ colleagues rated the experimental group significantly more competent than the control group to “quickly adapt to a new environment,” “establish a good doctor-patient relationship,” and “deal with on-call duty well” (Table 4).

**Table 4:** Colleague’s evaluations of the residents’ work performance

Question	*A*	*B*	*A* vs *B*
Familiar with hospital daily workflow	4.00(1.02)	3.96(1.04)	0.88
Able to quickly adapt to a new environment	4.39(0.92)	3.79(0.93)	< 0.05
Maintain good relationships with colleagues	4.21(0.69)	4.04(0.99)	0.47
Able to establish a good doctor-patient relationship	4.07(0.81)	3.54(0.980	< 0.05
Able to deal with on-call duty	4.14(0.65)	3.67(0.96)	< 0.05

*A*: Annual survey of experimental group, mean (standard deviation, SD); *B*: control group’s annual survey, mean (SD)

## Discussion

The “July effect” is a well-known phenomenon in which there is an increased incidence of adverse events during the first month of the training rotation of new residents because of their lack of experience [8, 9]. Therefore, there is a need to bridge the transition between medical school and residency by equipping novice residents with the appropriate surgical skills to “jump start” their careers and take advantage of early training opportunities in the hospital. Thus, novice residents in our hospital have been required since 2017 to participate in a two-week boot camp and a year of repetitive practice. This study has demonstrated that a surgical boot camp is an effective way to improve the theoretical knowledge and operating skills of new surgical residents and help them develop the confidence to deal with routine medical tasks. In addition, repetitive practice was found to be useful for residents to retain and strengthen their surgical skills and the confidence they acquired from the boot camp.

Medical boot camp provides residents the opportunity to obtain basic training to acquire and practice the requisite skills that are necessary for their future clinical work [10]. It provides residents with structured learning in an intensive and immersive environment that is useful for developing their skills and behaviors in controlled real-life scenarios. The distraction-free and stress-free simulation environment of boot camps is also useful because it relieves the tension of recruits while minimizing patient risk. A systematic review of 15 medical education boot camps indicated that participants in boot camp courses had significantly greater improvements in clinical skills and knowledge compared to control groups [11]. One study, which had a 5-day boot-camp training program, also found a remarkable increase in the confidence of trainees [12]. Our survey of the residents in the experimental group before and after boot camp also found residents had greater confidence to perform almost every medical task they were asked about after intensive training at boot camp, which further supported previous research findings.

Although boot camps can enable residents to acquire much-needed knowledge and skills in a short period of time, the camps’ effects diminish over time [12]. In addition, there is a delay between the time the skills are learned and the time the skills are needed in their daily medical work [13]. In order to overcome these shortcomings, several studies have recommended distributed practice and demonstrated the benefits of distributed practice over massed practice for learning surgical skills [14, 15]. The concept of distributed practice, referred to as a practice regime, entails periods of training that are interspersed with rest periods in order to repeat and reinforce skills [14]. It was similar to the concept of repetitive practice used in this study. When we learn a new skill or behavior, memory consolidation is needed, which is related to the characteristics of the learning curve. Repetitive practice can provide residents with more opportunities for consolidation. In addition, repetitive practice allows for cognitive preparation and mental rehearsal, which also help to strengthen memory [16]. Therefore, residents in the experimental group were required not only to participate in boot camp training, but also to do a year of repetitive practice, in order to take advantage of both training models. Our purpose was for them to enhance consolidation of what they had learned during boot camp, and though both groups received similar residency training during their first year, the residents in the experimental group seemed better qualified for residency training tasks.

The survey results showed that the self-confidence of residents increased significantly by the end of boot camp, except for the tasks “perform a surgical puncture” and “handle daily medical record writing.” Yet, it was encouraging to see that their level of confidence at the end of the boot camp was similar to that of the control group who had a full year of in-hospital training. This result is similar to that of a previous study [8]. Moreover, the confidence of the experimental group of residents further increased after a year of repetitive practice, and it was, in some respects, superior to that of the control group. Having the confidence to meet the challenges of clinical involvement early on was useful for them to master surgical skills better and ensure patient safety [17]. This also could explain why the residents in the experimental group received better evaluations from their colleagues.

## Limitations of the study

First, this was a comparison study with a small sample, which could not achieve enough power to do a statistical analysis. Therefore, larger randomized controlled trails are needed to make the conclusions more reliable. However, because this study was only a primary exploratory study, the ethical challenges are still large. Second, we did not compare the differences between boot camp and repetitive practice. This makes our findings somewhat ambiguous because we cannot distinguish between the effects boot camp and repetitive practice. Third, there is no research that has compared our training model with other training models. This is why we are going conduct another study. In a word, further studies are needed to better define the ideal training methods.

## Conclusion

This study showed that boot camp training is an effective way for residents to acquire surgical skills and confidence rapidly. Repetitive practice can further strengthen what they learn in boot camp. These findings have encouraged us to continue the surgical boot camp as well as repetitive practice in the future.

## Appendix

See Tables

**Table 5:** The boot camp curriculum

7/31	8/1	8/2	8/3	8/4
08:30–11:30 opening ceremony	08:30–10:00 thoracentesis	08:30–10:00 didactic lecture: How do residents teach medical students well?	08:30–12:00 live surgery: laparoscopic surgery on a animal	08:30–10:00 treatment of postoperative complications of gastrointestinal surgery
	10:30–12:00 cardiothoracic surgery teaching rounds	10:30–12:00 teaching rounds	14:30–15:45 diagnosis and treatment of perioperative dyspnea in cardiothoracic surgery	10:15–11:45 treatment of pseudoaneurysm hemorrhage or blood glucose critical value after pancreatectomy
	14:30–15:45 basic skills in laparoscopic surgery	14:30–15:45 simulation training: how to hand on-call emergencies	16:00–17:30 common surgical complications in hepatobiliary surgery	14:30–15:45 diagnosis of common emergency diseases in vascular surgery
	16:00–17:30 laparoscopic surgery simulation training	16:00–17:30 case discussion: acute abdomen		16:00–17:30 treatment of common emergency in neurosurgery
8/7	8/8	8/9	8/10	8/11
08:30–09:30 airway management in emergency situations	08:30–09:30 how to write medical records	08:30–10:00 surgical instrument identification and surgical sterility principles	08:30–12:00 simulation training: incision, suture, knot	08:30–12:00 surgical operation assessment
09:45–10:45 management of perioperative bleeding in abdominal surgery	09:45–10:45 management of burn patients	10:15–12:00 simulation training: surgical aseptic operation	14:30–17:30 simulation training: emergency debridement, suture hemostasis	14:00–15:00 theoretical examination
11:00–12:00 treatment of acute complications after thyroid surgery	11:00–12:00 urinary tract obstruction	14:30–15:45 place stomach tube		15:30–17:00 exchange of experience
14:30–15:45 emergency management of trauma patients	14:00–15:00 medical team communication skills	16:00–17:30 place the catheter		
16:00–17:30 identification and interpretation of clinical critical values	15:15–16:15 abdominal imaging (X-ray and CT)			
	16:30–17:30 surgical common catheter placement method			

5 and

**Table 6:** Scoring criteria for surgical dressing

Resident name: Examiner name: Total score:			
Item	Scoring criteria	SS	AS
Patient preparation	Understand the situation of the part to be changed and can evaluate the conditions that may occur during the operation (oral expression)	0.5	
	Inform the patient about the purpose of dressing change and the conditions that may occur during the process (oral expression)	0.5	
	Take appropriate posture of patients and protect the privacy of patients (oral expression)	0.5	
	Proper analgesia for complex wounds or when patients feel pain (oral expression)	0.5	
Operator preparation	Understand the patient's wound condition and assist the patient in placing the appropriate position (oral expression)	0.5	
	Choose a suitable dressing location, emphasize environmental sanitation, do not clean the dressing room half an hour before dressing change (oral expression)	0.5	
	Aseptic preparation by the operator: clothes mask, hat, hand washing, etc.	1	
Material preparation	1. Material: dressing bowl, plier, scissors, iodine volts or disinfectant alcohol, sterile cotton ball, dressing, tape, sterile gloves	1.5	
	2. Others: drainage material, probe, syringe, turpentine or gasoline, cotton swab, bandage, etc.	0.5	
Wound exposure	1. Uncover the outer dressing by hand and place it in the dressing plate	0.5	
	2. Gently peel the inner dressing with plier	0.5	
	3. If the wound adheres to the dressing, moisten it with saline and then uncover it	0.5	
Wound observation	1. Secretion traits	0.5	
	2. Observe the wound: redness, bleeding, suppuration, etc.	0.5	
	3. Observe the characteristics of skin, mucous membrane, and granulation tissue	0.5	
Wound disinfection	1. One plier is used to touch the wound, and the other is used to touch the sterile dressing. The two cannot be in direct contact	3	
	2. Hold the disinfecting cotton ball with the plier that touch wound and disinfect the wound three times from the inside-out	3	
Wound coverage	1. Covering at least 8 layers of dressing; the edge of the dressing is more than 3 cm from the wound	1	
	2. The direction of the tape is perpendicular to the long axis of the dressing. No tension	1	
Overall evaluation	1.Proficient in operating	1	
	2.Gentle operation	1	
	3. Aseptic consciousness	1	

SS: standard score; AS: actual score

6.
